# 2-Ethyl-3-hy­droxy-1-isopropyl-4-pyridone

**DOI:** 10.1107/S1600536812044091

**Published:** 2012-10-27

**Authors:** Pule P. Molokoane, M. Schutte, G. Steyl

**Affiliations:** a Department of Chemistry, University of Free State, Bloemfontein, 9301, PO Box 339, South Africa

## Abstract

The title compound, C_10_H_15_NO_2_, crystallized with three mol­ecules in the asymmetric unit. These three mol­ecules are quite similar except for slight differences in the torsion angles of the substituents on the ring. The isopropyl C—C—N—C torsion angles (towards the carbon next to the ethyl bound carbon), for example, are −150.63 (11), −126.77 (13) and −138.76 (11)° for mol­ecules *A*, *B* and *C*, respectively, and the C—C—C—N torsion angles involving the ethyl C atoms are 102.90 (13), 87.81 (14) and 86.47 (13)°. The main difference between the three mol­ecules lies in the way they are arranged in the solid-state structure. All three mol­ecules form dimers that are connected through strong O—H⋯O hydrogen bonds with *R*_2_^2^(10) graph-set motifs. The symmetry of the dimers formed does however differ between mol­ecules. Mol­ecules *B* connect with each other to form inversion dimers. Mol­ecules *A* and *C*, on the other hand, form dimers with local twofold symmetry, but the two mol­ecules are crystallographically distinct. The B and C molecules are linked to themselves and to each other *via* C—H⋯O hydrogen bonds. This results in the formation of a three-dimensional network structure.

## Related literature

For background on this type of ligand system, see: Fassihi *et al.* (2009 ▶); Weinberg (1994 ▶); Galanello, 2007 ▶); Scott *et al.* (2008 ▶). For similar structures, see: Xiao *et al.* (1992 ▶); Burgess *et al.* (1993 ▶); Hider *et al.* (1990 ▶); Dobbin *et al.* (1993 ▶); Brown *et al.* (1995 ▶).
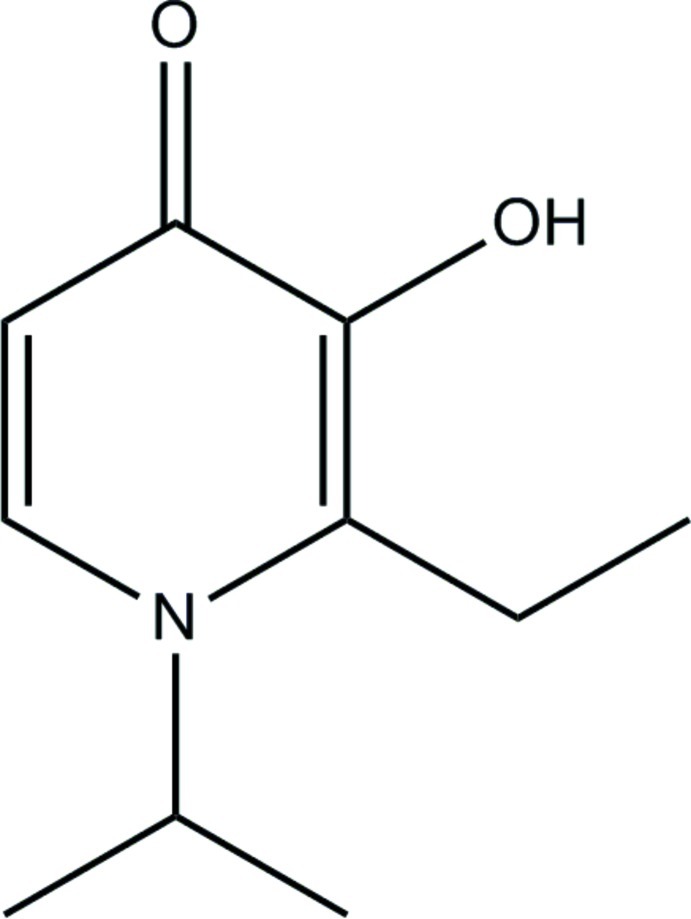


## Experimental

### 

#### Crystal data

C_10_H_15_NO_2_
*M* *_r_* = 181.23
Orthorhombic, 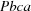

*a* = 11.7408 (2) Å
*b* = 13.3554 (2) Å
*c* = 37.5523 (8) Å
*V* = 5888.32 (18) Å^3^
*Z* = 24
Mo *K*α radiation
μ = 0.09 mm^−1^
*T* = 100 K
0.43 × 0.32 × 0.16 mm

#### Data collection

Bruker APEXII CCD diffractometer
Absorption correction: multi-scan (*SADABS*; Bruker, 2008 ▶) *T*_min_ = 0.968, *T*_max_ = 0.986
66795 measured reflections
7343 independent reflections
5939 reflections with *I* > 2σ(*I*)
*R*_int_ = 0.041

#### Refinement

*R*[*F* ^2^ > 2σ(*F* ^2^)] = 0.041
*wR*(*F* ^2^) = 0.111
*S* = 1.01
7343 reflections
373 parameters
H atoms treated by a mixture of independent and constrained refinement
Δρ_max_ = 0.31 e Å^−3^
Δρ_min_ = −0.28 e Å^−3^

### 

Data collection: *APEX2* (Bruker, 2008 ▶); cell refinement: *SAINT-Plus* (Bruker, 2008 ▶); data reduction: *SAINT-Plus*; program(s) used to solve structure: *SHELXS97* (Sheldrick, 2008 ▶); program(s) used to refine structure: *SHELXL97* (Sheldrick, 2008 ▶); molecular graphics: *DIAMOND* (Brandenburg & Putz, 2005 ▶); software used to prepare material for publication: *WinGX* (Farrugia, 1999 ▶).

## Supplementary Material

Crystal structure: contains datablock(s) global, I. DOI: 10.1107/S1600536812044091/zl2508sup1.cif

Structure factors: contains datablock(s) I. DOI: 10.1107/S1600536812044091/zl2508Isup2.hkl

Supplementary material file. DOI: 10.1107/S1600536812044091/zl2508Isup3.cml

Additional supplementary materials:  crystallographic information; 3D view; checkCIF report

## Figures and Tables

**Table 1:** Hydrogen-bond geometry (Å, °)

*D*—H⋯*A*	*D*—H	H⋯*A*	*D*⋯*A*	*D*—H⋯*A*
O2*A*—H2*A*⋯O1*C* ^i^	0.89 (2)	1.85 (2)	2.6503 (13)	149.9 (17)
O2*B*—H2*B*⋯O1*B* ^ii^	0.882 (19)	1.859 (18)	2.6480 (13)	147.8 (17)
O2*C*—H2*C*⋯O1*A* ^iii^	0.869 (18)	1.796 (18)	2.5868 (12)	150.3 (17)
C5*B*—H5*B*⋯O1*C* ^ii^	0.95	2.43	3.3237 (16)	156
C6*C*—H6*C*⋯O2*C* ^iv^	1.00	2.59	3.4623 (15)	146
C9*B*—H9*B*1⋯O1*B* ^v^	0.99	2.44	3.3548 (16)	153

Symmetry codes: (i) 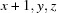; (ii) 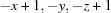; (iii) 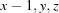; (iv) 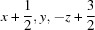; (v) 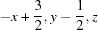.

**Fig. 1.**
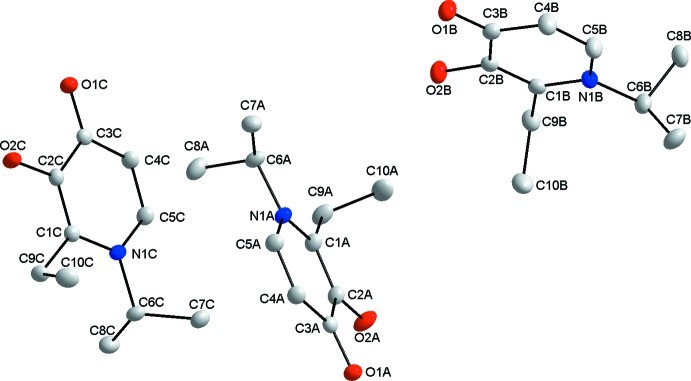
Representation of the title compound, showing the numbering scheme and displacement ellipsoids (50% probability). Hydrogen atoms were omitted for clarity.

**Fig. 2.**
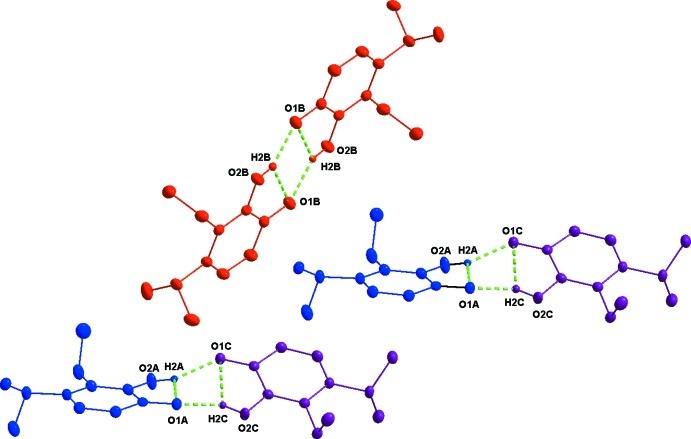
Hydrogen interactions (O—H···O) of the title compound in the crystal structure. (Molecule A in blue, molecule B in red and molecule C in purple).

**Fig. 3.**
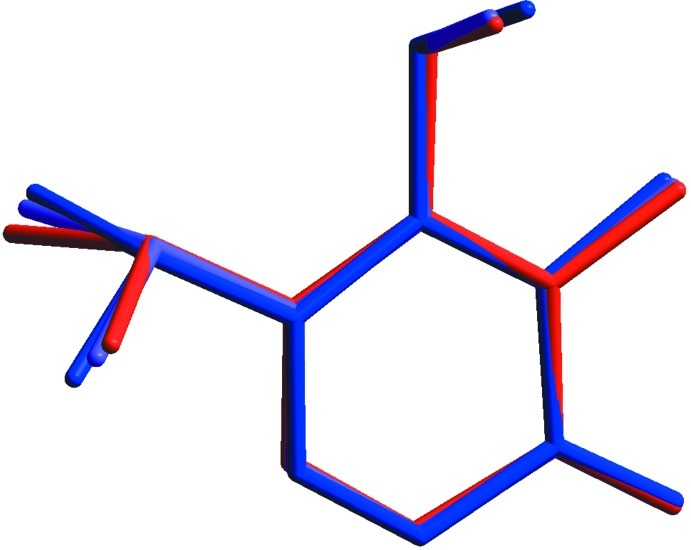
Least square overlay of all the atoms in the three independent molecules (Molecule A in blue, molecule B in red and molecule C in purple).

## supplementary crystallographic information

### Comment

3-Hydroxypyridinones, which are derivatives of 3-hydroxypyranones, are known to have antimicrobial and antimalarial activity (Fassihi *et al.*, 2009 and Weinberg, 1994). In addition to this, these compounds are non-toxic and they are approved for therapeutic use in some parts of the world (Galanello, 2007). Furthermore these organic compounds are metal ion chelators and they are used to prepare prodrugs with antioxidant characteristics and have brain targeting capabilities. These drugs have been suggested for the treatment of Alzheimer's disease and might possibly be more effective than treatments that just isolate metals (Scott *et al.*, 2008).

As part of an ongoing study, *O*,*O*'-donor bidentate ligands are obtained by functionalizing commercially available 3-hydroxy-2-methylpyran-4-one (maltol) and 3-hydroxy-2-ethylpyran-4-one (ethyl maltol) to the respective 3-hydroxy-2-methylpyrid-4-one and 3-hydroxy-2-ethylpyrid-4-one derivatives. The funtionalizations are performed in order to obtain an array of different electronic and steric properties imparted on the respective starting materials in order to study these effects. Coordination to copper(II) and designing a catalyst with a suitable support for oxidation and the kinetic study thereof are part of this study.

2-Ethyl-3-hydroxy-1-isopropylpyridinone crystallized in the orthorhombic *Pbca* space group with three molecules in the asymmetric unit. The average carbonyl distances (C=O) in the three molecules of 1.265 (4) Å are comparable to those of similar molecules that have been reported in the literature (Dobbin *et al.*, 1993, Xiao *et al.*, 1992, Burgess *et al.*, 1993, Hider *et al.*, 1990). These four structures differ only by the substituents on the N1 and C1 atoms and are reported as combinations of methyl and ethyl groups compared to ethyl (C1) and isopropyl (N1) for this structure. A distance of 1.265 (1) Å by Xiao *et al.* (1992), 1.275 (5) Å by Burgess *et al.* (1993), 1.271 (1) Å by Hider *et al.* (1990) and 1.264 (2) Å by Dobbin *et al.* (1993) have been reported. The three crystallograophically distinct molecules are quite similar, for instance the carbonyl distances for molecules A, B and C are 1.264 (1) Å, 1.261 (2) Å and 1.269 (1) Å respectively with r.m.s. values of 0.6447 Å (for an overlay of the complete molecule A and B), 0.6257 Å (for an overlay of the complete molecule B and C) and 0.1476 Å (for an overlay of the complete molecule A and C). Illustrated in Figure 3 is an overlay of all three molecules. As can be seen, the molecules are distinct by small variations in their torsion angles, C1—N1—C6—C7 and C10—C9—C1—N1. For molecule A, B and C respectively, C1—N1—C6—C7 and C10—C9—C1—N1 are -150.62 (11) °, -126.77 (13) ° and -138.76 (11) ° for the first and 102.90 (13) °, 87.81(154 ° and 86.47 (13) ° for the latter torsion angle. The main difference between the three molecules lies however in the way they are arranged in the solid state structure. All three molecules are forming dimers that are connected through strong O—H···O hydrogen bonds with graph set motifs of *R*_2_^2^(10). The symmetry of the dimers formed does however differ between molecules. Molecules B connect with each other to form dimers with exact crystallographic inversion symmetry. Molecules A and C, on the other hand form dimers with local two fold symmetry, but the two molecules are crystallographically distinct within the crystal lattice. Two weaker C—H···O intramolecular hydrogen interactions are formed between the ethyl carbon (C9) and the hydroxyl oxygen (O2) in molecule B and C. Another intramolecular C—H···O interactions is formed between the aromatic carbon C5B and a neighboring molecule's ketone oxygen (O1C). Finally, an intermolecular hydrogen interaction is observed between ethyl carbon C9B and a ketone oxygen (O1B).

### Experimental

2-Ethyl-3-hydroxy-1-isopropyl-4-pyridinone was prepared from the reflux of 2-ethyl-3-hydroxypyran-4-one (ethyl maltol) (5 g, 0,03568 mol) with 6 equivalents of aqueous isopropylamine (12.65 ml, 0,2141 mol, 99%) in 100 ml of water overnight. The mixture turned dark brown. Decolourizing charcoal was added after refluxing and the mixture was left to stand for 30 min. This was then filtered and the dark brown filtrate was evaporated *in vacuo* to yield a dark brown solid. Crystallization from cold acetone gave pink crystals of 2-ethyl-3-hydroxy-1-isopropyl-4-pyridinone (Yield - 2.5 g, 0.0138 mol, 50%). NMR (300 MHz)^13^C: 13.3, 18.5, 23.1, 51.5, 111.9, 133.4, 134.3, 145.2, 169.2. NMR (300 MHz) ^1^H: 1.11(*t*), 1.38(*d*), 2.77(*q*), 4.48(*m*), 6.18(*d*), 7.70(*d*).

### Refinement

Aromatic H atoms were positioned geometrically and allowed to ride on their parent atoms, with *U*_iso_(H) = 1.2*U*_eq_(parent) of the parent atom with a C—H distance of 0.93 Å. The methyl and methene H atoms were placed in geometrically idealized positions and constrained to ride on its parent atoms with *U*_iso_(H) = 1.5*U*_eq_(C) and *U*_iso_(H) = 1.2*U*_eq_(C) and at a distance of 0.96 Å and 0.97 Å respectively. The methine hydrogen atoms were placed in geometrically idealized positions and constrained to ride on its parent atoms with *U*_iso_(H) = 1.2 *U*_eq_(C) and at a distance of 0.98 Å. Hydroxyl H atoms were placed from the electron density map and refined freely. *U*_iso_(H) = 0.04216*U*_eq_(C) for molecule A, *U*_iso_(H) = 0.03874*U*_eq_(C) for molecule B and *U*_iso_(H) = 0.03682*U*_eq_(C) for molecule C.

### Crystal data

**Table:**

C_10_H_15_NO_2_	*F*(000) = 2352
*M**_r_* = 181.23	*D*_x_ = 1.227 Mg m^−^^3^*D*_m_ = 1.227 Mg m^−^^3^*D*_m_ measured by not measured
Orthorhombic, *P**b**c**a*	Mo *K*α radiation, λ = 0.71073 Å
Hall symbol: -P 2ac 2ab	Cell parameters from 9920 reflections
*a* = 11.7408 (2) Å	θ = 2.4–28.3°
*b* = 13.3554 (2) Å	µ = 0.09 mm^−^^1^
*c* = 37.5523 (8) Å	*T* = 100 K
*V* = 5888.32 (18) Å^3^	Cuboid, pink
*Z* = 24	0.43 × 0.32 × 0.16 mm

### Data collection

**Table:**

Bruker APEXII CCD diffractometer	5939 reflections with *I* > 2σ(*I*)
Graphite monochromator	*R*_int_ = 0.041
φ and ω scans	θ_max_ = 28.4°, θ_min_ = 2.1°
Absorption correction: multi-scan (*SADABS*; Bruker, 2008)	*h* = −15→15
*T*_min_ = 0.968, *T*_max_ = 0.986	*k* = −17→14
66795 measured reflections	*l* = −50→49
7343 independent reflections	

### Refinement

**Table:**

Refinement on *F*^2^	Primary atom site location: structure-invariant direct methods
Least-squares matrix: full	Secondary atom site location: difference Fourier map
*R*[*F*^2^ > 2σ(*F*^2^)] = 0.041	Hydrogen site location: inferred from neighbouring sites
*wR*(*F*^2^) = 0.111	H atoms treated by a mixture of independent and constrained refinement
*S* = 1.01	*w* = 1/[σ^2^(*F*_o_^2^) + (0.0549*P*)^2^ + 2.2867*P*] where *P* = (*F*_o_^2^ + 2*F*_c_^2^)/3
7343 reflections	(Δ/σ)_max_ = 0.001
373 parameters	Δρ_max_ = 0.31 e Å^−^^3^
0 restraints	Δρ_min_ = −0.28 e Å^−^^3^

### Special details

**Table:**

Geometry. All s.u.'s (except the s.u. in the dihedral angle between two l.s. planes) are estimated using the full covariance matrix. The cell s.u.'s are taken into account individually in the estimation of s.u.'s in distances, angles and torsion angles; correlations between s.u.'s in cell parameters are only used when they are defined by crystal symmetry. An approximate (isotropic) treatment of cell s.u.'s is used for estimating s.u.'s involving l.s. planes.
Refinement. Refinement of *F*^2^ against ALL reflections. The weighted *R*-factor *wR* and goodness of fit *S* are based on *F*^2^, conventional *R*-factors *R* are based on *F*, with *F* set to zero for negative *F*^2^. The threshold expression of *F*^2^ > 2σ(*F*^2^) is used only for calculating *R*-factors(gt) *etc*. and is not relevant to the choice of reflections for refinement. *R*-factors based on *F*^2^ are statistically about twice as large as those based on *F*, and *R*- factors based on ALL data will be even larger.

### Fractional atomic coordinates and isotropic or equivalent isotropic displacement parameters (Å^2^)

**Table:**

	*x*	*y*	*z*	*U*_iso_*/*U*_eq_	
O1A	0.91293 (7)	0.19653 (6)	0.67343 (2)	0.01841 (18)	
O2A	0.82821 (8)	0.01809 (6)	0.64712 (3)	0.0239 (2)	
N1A	0.59980 (8)	0.18817 (7)	0.62554 (3)	0.0163 (2)	
C1A	0.66341 (10)	0.10117 (8)	0.62811 (3)	0.0172 (2)	
C2A	0.76777 (10)	0.10457 (8)	0.64456 (3)	0.0169 (2)	
C3A	0.81506 (10)	0.19473 (9)	0.65940 (3)	0.0154 (2)	
C4A	0.74207 (10)	0.27933 (9)	0.65694 (3)	0.0180 (2)	
H4A	0.7658	0.3411	0.667	0.022*	
C5A	0.63923 (10)	0.27388 (9)	0.64048 (3)	0.0185 (2)	
H5A	0.5932	0.3323	0.6394	0.022*	
C6A	0.48310 (10)	0.18759 (9)	0.60952 (3)	0.0199 (3)	
H6A	0.4814	0.1348	0.5906	0.024*	
C7A	0.45372 (11)	0.28715 (10)	0.59208 (4)	0.0229 (3)	
H7A1	0.5154	0.3068	0.5759	0.034*	
H7A2	0.4442	0.3385	0.6105	0.034*	
H7A3	0.3827	0.2803	0.5786	0.034*	
C8A	0.39681 (11)	0.15899 (10)	0.63795 (4)	0.0275 (3)	
H8A1	0.4159	0.0929	0.6476	0.041*	
H8A2	0.3205	0.157	0.6274	0.041*	
H8A3	0.3985	0.2086	0.6572	0.041*	
C9A	0.62142 (11)	0.00365 (9)	0.61273 (4)	0.0227 (3)	
H9A1	0.5382	0.0083	0.6088	0.027*	
H9A2	0.6351	−0.0506	0.6302	0.027*	
C10A	0.67925 (13)	−0.02331 (11)	0.57770 (4)	0.0336 (3)	
H10G	0.6521	−0.0889	0.5696	0.05*	
H10H	0.7619	−0.0258	0.5812	0.05*	
H10I	0.661	0.0274	0.5597	0.05*	
O1B	0.56596 (7)	0.03944 (7)	0.46383 (3)	0.0233 (2)	
O2B	0.59291 (8)	−0.13800 (7)	0.50044 (3)	0.0222 (2)	
N1B	0.84051 (9)	−0.14085 (8)	0.44368 (3)	0.0189 (2)	
C1B	0.76037 (10)	−0.16947 (9)	0.46869 (3)	0.0175 (2)	
C2B	0.66930 (10)	−0.10833 (9)	0.47560 (3)	0.0165 (2)	
C3B	0.65095 (10)	−0.01527 (9)	0.45722 (3)	0.0181 (2)	
C4B	0.73506 (12)	0.00685 (10)	0.43113 (4)	0.0240 (3)	
H4B	0.7278	0.0662	0.4174	0.029*	
C5B	0.82553 (11)	−0.05457 (10)	0.42527 (4)	0.0233 (3)	
H5B	0.88	−0.0364	0.4077	0.028*	
C6B	0.94331 (11)	−0.20294 (10)	0.43529 (4)	0.0232 (3)	
H6B	0.9474	−0.2587	0.453	0.028*	
C7B	1.05136 (12)	−0.14100 (13)	0.43862 (5)	0.0384 (4)	
H7B1	1.0522	−0.107	0.4617	0.058*	
H7B2	1.0537	−0.0911	0.4195	0.058*	
H7B3	1.1179	−0.185	0.4367	0.058*	
C8B	0.93117 (13)	−0.24888 (10)	0.39849 (4)	0.0284 (3)	
H8B1	0.8596	−0.2864	0.3972	0.043*	
H8B2	0.9952	−0.2942	0.394	0.043*	
H8B3	0.9309	−0.1956	0.3805	0.043*	
C9B	0.77378 (11)	−0.26520 (9)	0.48918 (3)	0.0232 (3)	
H9B1	0.8115	−0.3158	0.4739	0.028*	
H9B2	0.6976	−0.2912	0.4957	0.028*	
C10B	0.84408 (13)	−0.24934 (12)	0.52279 (4)	0.0330 (3)	
H10D	0.848	−0.3121	0.5362	0.05*	
H10E	0.8083	−0.1976	0.5375	0.05*	
H10F	0.9212	−0.2281	0.5163	0.05*	
O1C	0.05201 (7)	−0.00440 (6)	0.65172 (2)	0.01955 (19)	
O2C	0.08600 (7)	0.12707 (7)	0.70817 (2)	0.02100 (19)	
N1C	0.33901 (8)	−0.02479 (7)	0.71342 (3)	0.0154 (2)	
C1C	0.25752 (10)	0.04654 (8)	0.72127 (3)	0.0152 (2)	
C2C	0.16221 (10)	0.05441 (8)	0.70010 (3)	0.0156 (2)	
C3C	0.14118 (10)	−0.01157 (8)	0.67065 (3)	0.0154 (2)	
C4C	0.22771 (10)	−0.08416 (9)	0.66494 (3)	0.0174 (2)	
H4C	0.2196	−0.1306	0.6459	0.021*	
C5C	0.32200 (10)	−0.08894 (8)	0.68600 (3)	0.0176 (2)	
H5C	0.3776	−0.1389	0.6813	0.021*	
C6C	0.44322 (10)	−0.03673 (9)	0.73602 (3)	0.0176 (2)	
H6C	0.4545	0.0272	0.7494	0.021*	
C7C	0.54870 (10)	−0.05396 (10)	0.71333 (4)	0.0223 (3)	
H7C1	0.5542	−0.0013	0.6952	0.033*	
H7C2	0.5433	−0.1194	0.7016	0.033*	
H7C3	0.6166	−0.0523	0.7285	0.033*	
C8C	0.42321 (11)	−0.11941 (10)	0.76313 (4)	0.0242 (3)	
H8C1	0.3555	−0.1036	0.7773	0.036*	
H8C2	0.4896	−0.1248	0.7788	0.036*	
H8C3	0.4116	−0.1831	0.7507	0.036*	
C9C	0.27357 (10)	0.11713 (8)	0.75202 (3)	0.0179 (2)	
H9C1	0.3138	0.0819	0.7715	0.021*	
H9C2	0.198	0.138	0.7611	0.021*	
C10C	0.34170 (12)	0.21007 (9)	0.74141 (4)	0.0240 (3)	
H10A	0.35	0.2542	0.7621	0.036*	
H10B	0.3015	0.2458	0.7224	0.036*	
H10C	0.4172	0.1898	0.7329	0.036*	
H2A	0.9000 (17)	0.0306 (14)	0.6530 (5)	0.042 (5)*	
H2B	0.5443 (15)	−0.0896 (14)	0.5054 (5)	0.039 (5)*	
H2C	0.0328 (15)	0.1317 (13)	0.6922 (5)	0.038 (5)*	

### Atomic displacement parameters (Å^2^)

**Table:**

	*U*^11^	*U*^22^	*U*^33^	*U*^12^	*U*^13^	*U*^23^
O1A	0.0164 (4)	0.0183 (4)	0.0205 (4)	0.0008 (3)	−0.0029 (3)	−0.0013 (3)
O2A	0.0166 (5)	0.0134 (4)	0.0417 (6)	0.0027 (3)	−0.0069 (4)	−0.0028 (4)
N1A	0.0144 (5)	0.0142 (4)	0.0204 (5)	0.0015 (4)	−0.0019 (4)	−0.0017 (4)
C1A	0.0169 (6)	0.0130 (5)	0.0217 (6)	0.0007 (4)	0.0008 (5)	−0.0010 (4)
C2A	0.0166 (6)	0.0126 (5)	0.0213 (6)	0.0012 (4)	0.0015 (4)	−0.0001 (4)
C3A	0.0161 (5)	0.0161 (5)	0.0139 (5)	−0.0009 (4)	0.0021 (4)	0.0011 (4)
C4A	0.0196 (6)	0.0134 (5)	0.0211 (6)	−0.0005 (4)	−0.0010 (5)	−0.0026 (4)
C5A	0.0201 (6)	0.0132 (5)	0.0222 (6)	0.0019 (4)	−0.0010 (5)	−0.0016 (4)
C6A	0.0159 (6)	0.0187 (5)	0.0252 (6)	0.0016 (4)	−0.0053 (5)	−0.0033 (5)
C7A	0.0227 (6)	0.0247 (6)	0.0213 (6)	0.0043 (5)	−0.0039 (5)	−0.0004 (5)
C8A	0.0183 (6)	0.0253 (6)	0.0388 (8)	0.0002 (5)	0.0000 (5)	0.0039 (6)
C9A	0.0179 (6)	0.0147 (5)	0.0355 (8)	0.0000 (5)	−0.0037 (5)	−0.0048 (5)
C10A	0.0332 (8)	0.0286 (7)	0.0391 (8)	−0.0024 (6)	−0.0033 (6)	−0.0161 (6)
O1B	0.0207 (4)	0.0217 (4)	0.0277 (5)	0.0048 (4)	0.0034 (4)	0.0038 (4)
O2B	0.0214 (5)	0.0177 (4)	0.0274 (5)	0.0010 (4)	0.0071 (4)	0.0033 (4)
N1B	0.0201 (5)	0.0194 (5)	0.0173 (5)	0.0037 (4)	0.0016 (4)	0.0005 (4)
C1B	0.0196 (6)	0.0173 (5)	0.0155 (6)	−0.0006 (4)	−0.0025 (4)	−0.0012 (4)
C2B	0.0174 (6)	0.0170 (5)	0.0149 (5)	−0.0022 (4)	−0.0014 (4)	−0.0013 (4)
C3B	0.0179 (6)	0.0177 (5)	0.0188 (6)	0.0001 (4)	−0.0023 (4)	−0.0011 (5)
C4B	0.0273 (7)	0.0211 (6)	0.0237 (6)	0.0041 (5)	0.0042 (5)	0.0058 (5)
C5B	0.0241 (6)	0.0243 (6)	0.0214 (6)	0.0027 (5)	0.0053 (5)	0.0048 (5)
C6B	0.0213 (6)	0.0252 (6)	0.0232 (6)	0.0080 (5)	0.0031 (5)	0.0017 (5)
C7B	0.0227 (7)	0.0444 (9)	0.0481 (10)	0.0044 (6)	−0.0057 (7)	−0.0040 (8)
C8B	0.0338 (7)	0.0235 (6)	0.0279 (7)	0.0047 (6)	0.0084 (6)	−0.0018 (5)
C9B	0.0277 (7)	0.0188 (6)	0.0232 (6)	0.0048 (5)	0.0043 (5)	0.0032 (5)
C10B	0.0335 (8)	0.0424 (8)	0.0232 (7)	0.0129 (7)	0.0007 (6)	0.0075 (6)
O1C	0.0160 (4)	0.0235 (4)	0.0192 (4)	0.0011 (3)	−0.0028 (3)	−0.0035 (4)
O2C	0.0180 (4)	0.0236 (4)	0.0214 (5)	0.0069 (3)	−0.0048 (4)	−0.0068 (4)
N1C	0.0144 (5)	0.0148 (4)	0.0170 (5)	−0.0002 (4)	−0.0019 (4)	−0.0004 (4)
C1C	0.0159 (5)	0.0139 (5)	0.0158 (5)	−0.0011 (4)	0.0010 (4)	0.0000 (4)
C2C	0.0158 (5)	0.0146 (5)	0.0164 (6)	0.0003 (4)	0.0019 (4)	−0.0004 (4)
C3C	0.0154 (5)	0.0157 (5)	0.0150 (5)	−0.0025 (4)	0.0014 (4)	0.0014 (4)
C4C	0.0176 (6)	0.0161 (5)	0.0184 (6)	−0.0017 (4)	−0.0004 (4)	−0.0033 (4)
C5C	0.0181 (6)	0.0131 (5)	0.0217 (6)	0.0005 (4)	0.0005 (5)	−0.0023 (4)
C6C	0.0152 (6)	0.0170 (5)	0.0207 (6)	0.0007 (4)	−0.0049 (4)	−0.0013 (5)
C7C	0.0161 (6)	0.0259 (6)	0.0249 (7)	−0.0006 (5)	−0.0023 (5)	0.0010 (5)
C8C	0.0226 (6)	0.0268 (6)	0.0231 (7)	0.0018 (5)	−0.0030 (5)	0.0051 (5)
C9C	0.0192 (6)	0.0170 (5)	0.0174 (6)	0.0009 (4)	−0.0027 (4)	−0.0035 (5)
C10C	0.0298 (7)	0.0171 (6)	0.0251 (7)	−0.0029 (5)	−0.0051 (5)	−0.0021 (5)

### Geometric parameters (Å, º)

**Table:**

O1A—C3A	1.2643 (14)	C6B—C7B	1.520 (2)
O2A—C2A	1.3589 (14)	C6B—H6B	1
O2A—H2A	0.89 (2)	C7B—H7B1	0.98
N1A—C5A	1.3563 (15)	C7B—H7B2	0.98
N1A—C1A	1.3846 (15)	C7B—H7B3	0.98
N1A—C6A	1.4965 (15)	C8B—H8B1	0.98
C1A—C2A	1.3729 (17)	C8B—H8B2	0.98
C1A—C9A	1.5077 (16)	C8B—H8B3	0.98
C2A—C3A	1.4383 (16)	C9B—C10B	1.523 (2)
C3A—C4A	1.4211 (16)	C9B—H9B1	0.99
C4A—C5A	1.3583 (17)	C9B—H9B2	0.99
C4A—H4A	0.95	C10B—H10D	0.98
C5A—H5A	0.95	C10B—H10E	0.98
C6A—C8A	1.5206 (19)	C10B—H10F	0.98
C6A—C7A	1.5218 (17)	O1C—C3C	1.2690 (14)
C6A—H6A	1	O2C—C2C	1.3543 (14)
C7A—H7A1	0.98	O2C—H2C	0.869 (18)
C7A—H7A2	0.98	N1C—C5C	1.3541 (15)
C7A—H7A3	0.98	N1C—C1C	1.3819 (15)
C8A—H8A1	0.98	N1C—C6C	1.4977 (14)
C8A—H8A2	0.98	C1C—C2C	1.3766 (16)
C8A—H8A3	0.98	C1C—C9C	1.5028 (16)
C9A—C10A	1.523 (2)	C2C—C3C	1.4356 (16)
C9A—H9A1	0.99	C3C—C4C	1.4205 (16)
C9A—H9A2	0.99	C4C—C5C	1.3619 (17)
C10A—H10G	0.98	C4C—H4C	0.95
C10A—H10H	0.98	C5C—H5C	0.95
C10A—H10I	0.98	C6C—C8C	1.5203 (18)
O1B—C3B	1.2614 (15)	C6C—C7C	1.5208 (17)
O2B—C2B	1.3533 (15)	C6C—H6C	1
O2B—H2B	0.882 (19)	C7C—H7C1	0.98
N1B—C5B	1.3552 (16)	C7C—H7C2	0.98
N1B—C1B	1.3834 (16)	C7C—H7C3	0.98
N1B—C6B	1.4978 (15)	C8C—H8C1	0.98
C1B—C2B	1.3702 (17)	C8C—H8C2	0.98
C1B—C9B	1.5003 (17)	C8C—H8C3	0.98
C2B—C3B	1.4378 (16)	C9C—C10C	1.5295 (17)
C3B—C4B	1.4221 (18)	C9C—H9C1	0.99
C4B—C5B	1.3600 (18)	C9C—H9C2	0.99
C4B—H4B	0.95	C10C—H10A	0.98
C5B—H5B	0.95	C10C—H10B	0.98
C6B—C8B	1.5188 (19)	C10C—H10C	0.98
			
C2A—O2A—H2A	110.7 (12)	C6B—C7B—H7B1	109.5
C5A—N1A—C1A	119.69 (10)	C6B—C7B—H7B2	109.5
C5A—N1A—C6A	118.90 (10)	H7B1—C7B—H7B2	109.5
C1A—N1A—C6A	121.16 (10)	C6B—C7B—H7B3	109.5
C2A—C1A—N1A	119.01 (10)	H7B1—C7B—H7B3	109.5
C2A—C1A—C9A	119.53 (10)	H7B2—C7B—H7B3	109.5
N1A—C1A—C9A	121.46 (10)	C6B—C8B—H8B1	109.5
O2A—C2A—C1A	118.02 (10)	C6B—C8B—H8B2	109.5
O2A—C2A—C3A	118.85 (10)	H8B1—C8B—H8B2	109.5
C1A—C2A—C3A	123.13 (10)	C6B—C8B—H8B3	109.5
O1A—C3A—C4A	124.05 (11)	H8B1—C8B—H8B3	109.5
O1A—C3A—C2A	121.89 (11)	H8B2—C8B—H8B3	109.5
C4A—C3A—C2A	114.06 (10)	C1B—C9B—C10B	111.30 (11)
C5A—C4A—C3A	121.53 (11)	C1B—C9B—H9B1	109.4
C5A—C4A—H4A	119.2	C10B—C9B—H9B1	109.4
C3A—C4A—H4A	119.2	C1B—C9B—H9B2	109.4
N1A—C5A—C4A	122.47 (11)	C10B—C9B—H9B2	109.4
N1A—C5A—H5A	118.8	H9B1—C9B—H9B2	108
C4A—C5A—H5A	118.8	C9B—C10B—H10D	109.5
N1A—C6A—C8A	109.20 (10)	C9B—C10B—H10E	109.5
N1A—C6A—C7A	112.09 (10)	H10D—C10B—H10E	109.5
C8A—C6A—C7A	111.75 (10)	C9B—C10B—H10F	109.5
N1A—C6A—H6A	107.9	H10D—C10B—H10F	109.5
C8A—C6A—H6A	107.9	H10E—C10B—H10F	109.5
C7A—C6A—H6A	107.9	C2C—O2C—H2C	111.8 (12)
C6A—C7A—H7A1	109.5	C5C—N1C—C1C	119.75 (10)
C6A—C7A—H7A2	109.5	C5C—N1C—C6C	118.95 (10)
H7A1—C7A—H7A2	109.5	C1C—N1C—C6C	121.20 (9)
C6A—C7A—H7A3	109.5	C2C—C1C—N1C	119.49 (10)
H7A1—C7A—H7A3	109.5	C2C—C1C—C9C	119.85 (10)
H7A2—C7A—H7A3	109.5	N1C—C1C—C9C	120.64 (10)
C6A—C8A—H8A1	109.5	O2C—C2C—C1C	117.56 (10)
C6A—C8A—H8A2	109.5	O2C—C2C—C3C	119.91 (10)
H8A1—C8A—H8A2	109.5	C1C—C2C—C3C	122.53 (10)
C6A—C8A—H8A3	109.5	O1C—C3C—C4C	123.85 (11)
H8A1—C8A—H8A3	109.5	O1C—C3C—C2C	121.81 (10)
H8A2—C8A—H8A3	109.5	C4C—C3C—C2C	114.33 (10)
C1A—C9A—C10A	112.91 (11)	C5C—C4C—C3C	121.72 (11)
C1A—C9A—H9A1	109	C5C—C4C—H4C	119.1
C10A—C9A—H9A1	109	C3C—C4C—H4C	119.1
C1A—C9A—H9A2	109	N1C—C5C—C4C	122.11 (11)
C10A—C9A—H9A2	109	N1C—C5C—H5C	118.9
H9A1—C9A—H9A2	107.8	C4C—C5C—H5C	118.9
C9A—C10A—H10G	109.5	N1C—C6C—C8C	109.31 (10)
C9A—C10A—H10H	109.5	N1C—C6C—C7C	111.33 (10)
H10G—C10A—H10H	109.5	C8C—C6C—C7C	113.03 (10)
C9A—C10A—H10I	109.5	N1C—C6C—H6C	107.6
H10G—C10A—H10I	109.5	C8C—C6C—H6C	107.6
H10H—C10A—H10I	109.5	C7C—C6C—H6C	107.6
C2B—O2B—H2B	111.2 (12)	C6C—C7C—H7C1	109.5
C5B—N1B—C1B	119.54 (10)	C6C—C7C—H7C2	109.5
C5B—N1B—C6B	117.91 (10)	H7C1—C7C—H7C2	109.5
C1B—N1B—C6B	122.53 (10)	C6C—C7C—H7C3	109.5
C2B—C1B—N1B	119.65 (11)	H7C1—C7C—H7C3	109.5
C2B—C1B—C9B	119.51 (11)	H7C2—C7C—H7C3	109.5
N1B—C1B—C9B	120.81 (11)	C6C—C8C—H8C1	109.5
O2B—C2B—C1B	118.24 (11)	C6C—C8C—H8C2	109.5
O2B—C2B—C3B	118.99 (10)	H8C1—C8C—H8C2	109.5
C1B—C2B—C3B	122.76 (11)	C6C—C8C—H8C3	109.5
O1B—C3B—C4B	124.36 (11)	H8C1—C8C—H8C3	109.5
O1B—C3B—C2B	121.66 (11)	H8C2—C8C—H8C3	109.5
C4B—C3B—C2B	113.97 (11)	C1C—C9C—C10C	111.99 (10)
C5B—C4B—C3B	121.90 (12)	C1C—C9C—H9C1	109.2
C5B—C4B—H4B	119	C10C—C9C—H9C1	109.2
C3B—C4B—H4B	119	C1C—C9C—H9C2	109.2
N1B—C5B—C4B	122.12 (12)	C10C—C9C—H9C2	109.2
N1B—C5B—H5B	118.9	H9C1—C9C—H9C2	107.9
C4B—C5B—H5B	118.9	C9C—C10C—H10A	109.5
N1B—C6B—C8B	109.83 (11)	C9C—C10C—H10B	109.5
N1B—C6B—C7B	110.73 (11)	H10A—C10C—H10B	109.5
C8B—C6B—C7B	111.90 (12)	C9C—C10C—H10C	109.5
N1B—C6B—H6B	108.1	H10A—C10C—H10C	109.5
C8B—C6B—H6B	108.1	H10B—C10C—H10C	109.5
C7B—C6B—H6B	108.1		
			
C5A—N1A—C1A—C2A	−2.65 (17)	O1B—C3B—C4B—C5B	179.55 (13)
C6A—N1A—C1A—C2A	−176.91 (11)	C2B—C3B—C4B—C5B	−1.61 (19)
C5A—N1A—C1A—C9A	178.40 (12)	C1B—N1B—C5B—C4B	1.62 (19)
C6A—N1A—C1A—C9A	4.14 (18)	C6B—N1B—C5B—C4B	−179.73 (12)
N1A—C1A—C2A—O2A	179.30 (11)	C3B—C4B—C5B—N1B	0.6 (2)
C9A—C1A—C2A—O2A	−1.73 (18)	C5B—N1B—C6B—C8B	−69.46 (15)
N1A—C1A—C2A—C3A	−0.05 (18)	C1B—N1B—C6B—C8B	109.15 (13)
C9A—C1A—C2A—C3A	178.92 (11)	C5B—N1B—C6B—C7B	54.63 (16)
O2A—C2A—C3A—O1A	2.90 (18)	C1B—N1B—C6B—C7B	−126.77 (13)
C1A—C2A—C3A—O1A	−177.75 (11)	C2B—C1B—C9B—C10B	−90.30 (14)
O2A—C2A—C3A—C4A	−176.74 (11)	N1B—C1B—C9B—C10B	87.84 (14)
C1A—C2A—C3A—C4A	2.61 (17)	C5C—N1C—C1C—C2C	−2.87 (16)
O1A—C3A—C4A—C5A	177.72 (12)	C6C—N1C—C1C—C2C	−179.21 (10)
C2A—C3A—C4A—C5A	−2.65 (17)	C5C—N1C—C1C—C9C	178.76 (11)
C1A—N1A—C5A—C4A	2.66 (18)	C6C—N1C—C1C—C9C	2.42 (16)
C6A—N1A—C5A—C4A	177.05 (11)	N1C—C1C—C2C—O2C	−178.26 (10)
C3A—C4A—C5A—N1A	0.14 (19)	C9C—C1C—C2C—O2C	0.12 (16)
C5A—N1A—C6A—C8A	−89.31 (13)	N1C—C1C—C2C—C3C	2.50 (17)
C1A—N1A—C6A—C8A	84.99 (13)	C9C—C1C—C2C—C3C	−179.13 (11)
C5A—N1A—C6A—C7A	35.08 (15)	O2C—C2C—C3C—O1C	−0.37 (17)
C1A—N1A—C6A—C7A	−150.61 (11)	C1C—C2C—C3C—O1C	178.86 (11)
C2A—C1A—C9A—C10A	−76.04 (16)	O2C—C2C—C3C—C4C	179.80 (10)
N1A—C1A—C9A—C10A	102.90 (14)	C1C—C2C—C3C—C4C	−0.97 (16)
C5B—N1B—C1B—C2B	−2.64 (17)	O1C—C3C—C4C—C5C	−179.98 (11)
C6B—N1B—C1B—C2B	178.78 (11)	C2C—C3C—C4C—C5C	−0.15 (17)
C5B—N1B—C1B—C9B	179.23 (12)	C1C—N1C—C5C—C4C	1.82 (17)
C6B—N1B—C1B—C9B	0.65 (17)	C6C—N1C—C5C—C4C	178.24 (11)
N1B—C1B—C2B—O2B	−179.43 (10)	C3C—C4C—C5C—N1C	−0.29 (18)
C9B—C1B—C2B—O2B	−1.27 (17)	C5C—N1C—C6C—C8C	−80.72 (13)
N1B—C1B—C2B—C3B	1.54 (18)	C1C—N1C—C6C—C8C	95.65 (12)
C9B—C1B—C2B—C3B	179.70 (11)	C5C—N1C—C6C—C7C	44.86 (14)
O2B—C2B—C3B—O1B	0.40 (18)	C1C—N1C—C6C—C7C	−138.77 (11)
C1B—C2B—C3B—O1B	179.42 (12)	C2C—C1C—C9C—C10C	−91.88 (13)
O2B—C2B—C3B—C4B	−178.47 (11)	N1C—C1C—C9C—C10C	86.48 (13)
C1B—C2B—C3B—C4B	0.55 (17)		

### Hydrogen-bond geometry (Å, º)

**Table:**

*D*—H···*A*	*D*—H	H···*A*	*D*···*A*	*D*—H···*A*
O2*A*—H2*A*···O1*C*^i^	0.89 (2)	1.85 (2)	2.6503 (13)	149.9 (17)
O2*B*—H2*B*···O1*B*^ii^	0.882 (19)	1.859 (18)	2.6480 (13)	147.8 (17)
O2*C*—H2*C*···O1*A*^iii^	0.869 (18)	1.796 (18)	2.5868 (12)	150.3 (17)
C5*B*—H5*B*···O1*C*^ii^	0.95	2.43	3.3237 (16)	156
C6*C*—H6*C*···O2*C*^iv^	1	2.59	3.4623 (15)	146
C9*B*—H9*B*1···O1*B*^v^	0.99	2.44	3.3548 (16)	153

Symmetry codes: (i) *x*+1, *y*, *z*; (ii) −*x*+1, −*y*, −*z*+1; (iii) *x*−1, *y*, *z*; (iv) *x*+1/2, *y*, −*z*+3/2; (v) −*x*+3/2, *y*−1/2, *z*.
